# Differences in characteristics and use of complementary and alternative methods for coping with endometriosis-associated acyclic pelvic pain across adolescence and adulthood

**DOI:** 10.3389/frph.2023.1306380

**Published:** 2024-01-08

**Authors:** Jennifer M. Mongiovi, Britani Wallace, McKenzie Goodwin, Allison F. Vitonis, Sarah Karevicius, Amy L. Shafrir, Naoko Sasamoto, Amy D. DiVasta, Christine B. Sieberg, Kathryn L. Terry, Stacey A. Missmer

**Affiliations:** ^1^ Department of Epidemiology, Harvard T. H. Chan School of Public Health, Boston, MA, United States; ^2^ Department of Obstetrics, Gynecology, and Reproductive Biology, Brigham and Women’s Hospital and Harvard Medical School, Boston, MA, United States; ^3^ Boston Center for Endometriosis, Boston Children’s Hospital and Brigham and Women’s Hospital, Boston, MA, United States; ^4^ Division of Adolescent and Young Adult Medicine, Department of Pediatrics, Boston Children’s Hospital and Harvard Medical School, Boston, MA, United States; ^5^ Department of Nutrition and Public Health, School of Nursing and Health Sciences, Merrimack College, North Andover, MA, United States; ^6^ Biobehavioral Pain Innovations Lab, Department of Psychiatry & Behavioral Sciences, Boston Children’s Hospital, Boston, MA, United States; ^7^ Pain & Affective Neuroscience Center, Department of Anesthesiology, Critical Care, & Pain Medicine, Boston Children’s Hospital, Boston, MA, United States; ^8^ Department of Psychiatry, Harvard Medical School, Boston, MA, United States; ^9^ Department of Obstetrics, Gynecology, and Reproductive Biology, College of Human Medicine, Michigan State University, Grand Rapids, MI, United States

**Keywords:** endometriosis, adolescent, pelvic pain, complementary therapies, dysmenorrhea, pain triggers, pain remediators

## Abstract

**Introduction:**

Over four million women in the US alone have been diagnosed with endometriosis. For those living with this disease, surgery and hormonal treatment reduce associated pelvic pain in some, while others continue to experience life impacting pain. Therefore, identification of accessible and cost-effective methods of pain reduction to compliment current treatment is urgently needed. Our objective was to quantify the prevalence of complementary and alternative methods used to manage acyclic pelvic pain and their reported benefit among women of different age groups living with endometriosis.

**Methods:**

We used baseline questionnaire data from laparoscopically-confirmed endometriosis cases who completed a WERF EPHect compliant questionnaire in the longitudinal cohort of The Women's Health Study: From Adolescence to Adulthood (A2A). Participants with acyclic pelvic pain were asked to indicate specific methods or activities that either helped or worsened their pelvic/lower abdominal pain. Differences among age groups [adolescent (<18 years), young adult (18–25 years), and adult (>25 years)] were assessed using Fisher's exact test.

**Results:**

Of the 357 participants included in analysis, sleep for coping was reported more frequently among adolescents (*n* = 59, 57.3%) compared to young adults (*n* = 40, 44.0%) and adults (*n* = 19, 31.1%; *p* = 0.004). Adolescents also reported more frequent use of music (*n* = 29, 21.2%) than young adults (*n* = 10, 7.0%) and adults (*n* = 7, 9.1%; *p* = 0.001). Exercise worsened pain most commonly among adolescents (*n* = 82, 59.9%), followed by younger adults (*n* = 67, 46.9%), and adults (*n* = 27, 35.1%; *p* = 0.002).

**Discussion:**

Our analysis of participants in the A2A cohort showed that the prevalence of complementary and alternative methods used for coping with endometriosis-associated acyclic pelvic pain varied by age group. Future studies should aim to provide information that will further inform decisions in making care plans for managing endometriosis-associated pain that is effective, accessible, and tailored to the preferences of the patient.

## Introduction

Endometriosis is a chronic inflammatory condition that affects approximately 190 million women worldwide (1), although the true prevalence remains largely unknown due to variability in diagnostic approaches and the population of interest (2). A definitive diagnosis can take 5–10 years (3) and severity of symptoms is not directly related to the revised Society of Reproductive Medicine (rASRM) staging classification (4). Symptoms vary greatly, but often include dysmenorrhea, acyclic pelvic pain, dyspareunia, and dyschezia, all of which contribute to chronic general pelvic/lower abdominal pain that can continue from menarche through menopause (1). Current standard of care for the primary endometriosis symptom of persistent pelvic/lower abdominal pain consists of surgery (excision or ablation of lesions), use of hormonal medication, and management with over the counter (OTC) pain medications (ibuprofen, acetaminophen, naproxen) (5). Although these therapies are effective for some, they are not always sufficient to eliminate a patient's pain entirely and can potentially exacerbate some symptoms (6), especially among younger women (7, 8). In addition, all three treatments have varying degrees of accompanying risks or side effects that further vary with age.

Women with endometriosis-related pain often seek out strategies to augment or supplement these standard surgical, hormonal, and analgesic treatments. Acyclic pelvic pain, or pelvic pain occurring at times other than with menses, can be challenging to manage since it is associated with flares that are much less predictable than other symptoms. While some patients seek recommendations from their health care provider(s), there are no established pelvic pain management protocols or decision trees (3). Many studies of alternative methods for reducing endometriosis-associated pain included only single or similar strategies assessed in each study or included a small number of cases, leaving the need for a more comprehensive assessment among a greater number of women with endometriosis. Furthermore, how adolescents and young adults approach managing chronic pain may be different, and often the pain management plans for adults may not be appropriate for these younger age groups (9).

There exists a need for the identification of accessible, cost-effective, and age-appropriate methods of pain reduction to complement current surgical and hormonal treatment. Therefore, our objective was to quantify the prevalence of complementary and alternative methods used and their reported benefit among adolescent, young adult, and adult participants with endometriosis who reported continued acyclic pelvic pain after surgery.

## Methods

### Study design

The Women's Health Study: From Adolescence to Adulthood (A2A) is a longitudinal cohort that enrolled girls and women from 2012 to 2018, oversampling for those surgically diagnosed with endometriosis (10). Endometriosis cases were identified from Boston Children's Hospital (BCH) and Brigham and Women's Hospital (BWH) and were eligible for enrollment in the A2A if they were female, between age 7 and 55, and had laparoscopically-confirmed endometriosis. Participants were categorized as adults (age ≥25), young adults (age 18–24), and adolescents (age <18), based on the 2018 World Health Organization definition of adolescence (11).

At baseline and annual follow-up, eligible participants were sent a link to the REDCap survey via email or mailed a paper copy (12, 13). Questionnaires included extensive questions on behavioral and reproductive factors, as well as endometriosis-specific questions regarding pelvic pain, including severity, frequency, and pain interference. Questionnaires collected after January 2014 were World Endometriosis Research Foundation Endometriosis Phenome and Biobanking Harmonization Project (WERF EPHect) compliant (14). This study was approved by the Boston Children's Hospital Institutional Review Board. All participants provided written consent, with parental consent plus participant assent for girls younger than 18 years.

### Study variables

Participants were asked to indicate whether any of 18 listed methods provided relief from acyclic pelvic pain. These methods included pain medication, relaxation, lying down, music, massage, hot bath, ice, heating pad, bowel movement, urination, laxative/enema, meditation, injection, TENS unit, and other unspecified. In May 2013, yoga, physical activity, acupuncture, and sleep were added to the questionnaire. Participants were also asked to indicate whether no interventions helped their pain. Additionally, participants were asked what makes acyclic pelvic pain worse. The 17 options included sitting, standing, stress, full meal, bowel movement, constipation, full bladder, urination, walking, exercise, time of day, coughing/sneezing, intercourse, orgasm, other, as well as weather and contact with clothing, which were added to the questionnaire when the WERF EPHect compliant survey was adopted in 2014.

Demographic and anthropometric variables included age at baseline (years), race (Black, White, other/unknown), Hispanic origin (yes, no), school or work status (current middle/high school student, current college/graduate school student, working and not in school, other), ever smoked more than 100 cigarettes during lifetime (yes, no), and body mass index (BMI). For women aged 20 and older, BMI was categorized according to the World Health Organization Criteria: underweight (BMI <18.5 kg/m^2^), healthy weight (BMI 18.5–24.9 kg/m^2^), overweight (BMI 25–29.9 kg/m^2^), or obese (BMI ≥30 kg/m^2^). For those less than 20 years, the age- and gender-specific BMI Z-score was calculated, and participants were categorized as underweight (Z-score ≤ −2), healthy weight (Z-score > −2 to <1), overweight (Z-score 1–2), or obese (Z-score > 2). Medical and reproductive history included nulliparous (yes, no), ever used hormonal medications (yes, no), regular analgesic use ≥2 days a week for 3 months or longer (yes, no), age at menarche (years), family history of endometriosis (yes, no), period pain onset relative to menarche (had pain at first period, within 2 years of first period, more than 2 years after first period), usual period pain (no pain, mild cramps, moderate cramps, severe cramps, not cycling), and number of other pain conditions (e.g., lupus, rheumatoid arthritis, Crohn's disease; 0, 1, ≥2).

Additional endometriosis-specific characteristics included age at endometriosis symptom onset (years), years between symptom onset and surgical diagnosis, symptoms prompting diagnosis (pain only, infertility, or other conditions with or without pain), number of physicians seen before surgical diagnosis, rASRM stage at surgery (I/II, III/IV), and macrophenotype at surgery (superficial only, endometrioma, deep lesions, deep lesions and endometrioma). Characteristics of acyclic pelvic pain over the last 3 months included severity of acyclic pelvic pain using the 0 to 10 numeric rating scale (mild: 1–3, moderate: 4–6, and severe: 7–10), frequency of acyclic pelvic pain (<1 day per month, monthly but not weekly, weekly but not daily, daily), pain interfered with work or school (yes, no), pain medications taken (none, over the counter only, prescription only, prescription and over the counter), hormonal medications used (none, yes but pain did not get better, yes and pain got somewhat better), and narcotic prescription pain medication use for ≥3 months (yes, no).

### Statistical analysis

Of the 583 participants with laparoscopically-confirmed endometriosis who completed the baseline questionnaire, *n* = 219 who did not report experiencing acyclic pelvic pain within the past three months and *n* = 7 who did not complete the pain questionnaire were excluded. Using cross-sectional data from baseline questionnaires, frequencies for each method used to alleviate pain were calculated, overall and by age range defined as adolescent (<18 years), young adult (18–25 years), and adult (>25 years). Fisher's exact test was used to determine whether traditional pain management, complementary methods for lowering general pelvic/lower abdominal pain, and activities and conditions that worsen pelvic pain varied by age group. All statistical analyses were performed using SAS version 9.4 (SAS Institute Inc., Cary, NC).

## Results

A total of 357 adolescents, young adults, and adults with laparoscopically-confirmed endometriosis reported acyclic pelvic pain in the last 3 months (Table 1). The median age of participants at enrollment was 19 years [interquartile range (IQR): 16–24]. Most study participants were White (*n* = 326, 91.3%) and non-Hispanic (*n* = 335, 93.8%). At the time of questionnaire completion, the largest proportion of study participants were current middle or high school students (*n* = 126, 35.3%), healthy weight (*n* = 220, 61.6%), never smoked cigarettes (*n* = 323, 96.3%), were never pregnant (*n* = 333, 93.5%), ever used hormonal medications (*n* = 349, 97.8%), had a family history of endometriosis (*n* = 186, 52.1%), had pain at first period (*n* = 164, 48.2%), had no period in the last three months (*n* = 156, 43.9%), and had two or more other pain conditions (*n* = 256, 72.3%). Adolescents (age <18) were more likely to be healthy weight (*n* = 102, 74.5%), have a family history of endometriosis (*n* = 78, 56.9%), have pain at first period (*n* = 76, 56.7%), have severe period cramps (*n* = 59, 43.1%), and not have other pain conditions (*n* = 11, 8.0%) compared to the older age groups.

**Table 1:** Baseline demographic, anthropometric, medical history, and reproductive characteristics among A2A participants with laparoscopically confirmed endometriosis who reported experiencing acyclic pelvic pain in the last 3 months at baseline (*n* = 357).

	All cases *N* = 357	Adolescent (age <18) *N* = 137	Young Adult (age 18–25) *N* = 143	Adult (age >25) *N* = 77
Demographic and anthropometric				
Age at baseline, *y*, median (IQR)	19 (16–24)	16 (15–17)	21 (19–23)	30 (27–36)
Race, *n* (%)				
Black	11 (3.1%)	5 (3.6%)	2 (1.4%)	4 (5.2%)
White	326 (91.3%)	122 (89.1%)	134 (93.7%)	70 (90.9%)
Other/unknown^a^	20 (5.6%)	10 (7.3%)	7 (4.9%)	3 (3.9%)
Non-Hispanic ethnicity, *n* (%)	335 (93.8%)	127 (92.7%)	137 (95.8%)	71 (92.2%)
School or work status, *n* (%)				
Current middle/high school student	126 (35.3%)	119 (86.9%)	7 (4.9%)	0 (0%)
Current college/graduate school student	81 (22.7%)	2 (1.5%)	70 (49.0%)	9 (11.7%)
Working, not in school	112 (31.4%)	13 (9.5%)	46 (32.2%)	53 (68.8%)
Other	38 (10.6%)	3 (2.2%)	20 (14.0%)	15 (19.5%)
Never smoked, *n* (%)	323 (95.3%)	129 (99.2%)	131 (97.0%)	63 (85.1%)
Body mass index^b^, *n* (%)				
Underweight	6 (1.7%)	1 (0.7%)	5 (3.5%)	0 (0%)
Healthy weight	220 (61.6%)	102 (74.5%)	85 (59.4%)	33 (42.9%)
Overweight	85 (23.8%)	28 (20.4%)	36 (25.2%)	21 (27.3%)
Obese	46 (12.9%)	6 (4.4%)	17 (11.9%)	23 (29.9%)
Medical and reproductive				
Nulliparous, *n* (%)	333 (93.5%)	137 (100.0%)	141 (99.3%)	55 (71.4%)
Ever used hormonal medications, *n* (%)	349 (97.8%)	130 (94.9%)	143 (100.0%)	76 (98.7%)
Regular analgesic use (≥2 days/week)^c^, *n* (%)	163 (46.0%)	65 (47.4%)	56 (39.7%)	42 (55.3%)
Age at menarche, *y*, median (IQR)	12 (11–13)	12 (11–13)	12 (11–13)	12 (11–13)
Family history of endometriosis, *n* (%)	186 (52.1%)	78 (56.9%)	70 (49.0%)	38 (49.4%)
Period pain onset relative to menarche, *n* (%)				
Had pain at first period	164 (48.2%)	76 (56.7%)	61 (45.2%)	27 (38.0%)
Within 2 years of first period	114 (33.5%)	43 (32.1%)	48 (35.6%)	23 (32.4%)
More than 2 years after first period	62 (18.2%)	15 (11.2%)	26 (19.3%)	21 (29.6%)
Period pain, *n* (%)				
No pain	3 (0.8%)	1 (0.7%)	2 (1.4%)	0 (0%)
Mild cramps	12 (3.4%)	5 (3.6%)	3 (2.1%)	4 (5.2%)
Moderate cramps	51 (14.4%)	28 (20.4%)	13 (9.2%)	10 (13.0%)
Severe cramps	133 (37.5%)	59 (43.1%)	44 (31.2%)	30 (39.0%)
No periods in the last three months	156 (43.9%)	44 (32.1%)	79 (56.0%)	33 (42.9%)
Number of other pain conditions^d^, *n* (%)				
0	22 (6.2%)	11 (8.0%)	7 (5.0%)	4 (5.3%)
1	76 (21.5%)	32 (23.4%)	31 (22.0%)	13 (17.1%)
≥2	256 (72.3%)	94 (68.6%)	103 (73.0%)	59 (77.6%)

IQR, interquartile range; A2A, The Women's Health Study: From Adolescence to Adulthood.

^a^ Participants in the other/unknown category include American Indian/Alaska Native (*n* = 1), multiracial (*n* = 11), other race (*n* = 7), and unknown (*n* = 1).

^b^ For women aged ≥20 years: underweight [body mass index (BMI) < 18.5 kg/m^2^], normal weight (BMI 18.5–24.9 kg/m^2^), overweight (BMI 25–29.9 kg/m^2^), or obese (BMI ≥ 30 kg/m^2^) according to World Health Organization criteria. For those <20 years, the age-specific and gender-specific BMI Z-score was calculated and participants were categorized as underweight (Z-score ≤ −2), normal weight (Z-score > −2 to <1), overweight (Z-score 1–2), or obese (Z-score > 2).

^c^ Regular use of analgesic medications defined as use at least once a week for a period of 3 months or longer and categorized as never, <2 days of use per week, or ≥2 days of use per week. Analgesic medications include aspirin, acetaminophen, nonsteroidal anti-inflammatory drugs, and narcotics.

^d^ Includes migraine, low back pain, bladder pain, interstitial cystitis/painful bladder syndrome, chronic fatigue syndrome, fibromyalgia, irritable bowel syndrome, ulcerative colitis, Crohn disease, lupus, and rheumatoid arthritis.

The median age at endometriosis symptom onset was 14 years (IQR: 12–16) with 2 years (IQR: 1–5) of symptoms and 3 physicians (IQR: 2–4) were seen before surgical diagnosis. The majority of participants reported having superficial endometriosis (*n* = 291, 95.1%) and rASRM stage I/II disease (*n* = 277, 94.9%) (Table 2). Acyclic pelvic pain severity was most commonly severe (*n* = 214, 69.5%), occurred weekly (*n* = 135, 38.0%), and interfered with work or school (*n* = 236, 67.8%). A total of 159 participants (77.2%) used pain medication within the last three months to manage their acyclic pelvic pain. Almost half of participants reported using over the counter medications (*n* = 95, 46.1%) and about 30% of participants reported using prescription medications with or without over-the-counter medications (*n* = 64, 31.1%), and less then 10% of participants used narcotic prescription pain medications for 3 months or longer (*n* = 16, 7.5%). Adolescents were more likely to be diagnosed with rASRM Stage I/II (*n* = 126, 98.4%), report interference of acyclic pelvic pain with work or school (*n* = 100, 75.2%), and only used over-the-counter medications for pain (*n* = 40, 48.2%) compared to older age groups.

**Table 2:** Distribution of endometriosis and acyclic pelvic pain characteristics among A2A participants with laparoscopically confirmed endometriosis who reported experiencing acyclic pelvic pain in the last 3 months at baseline (*n* = 357).

	All cases *N* = 357	Adolescent (age <18) *N* = 137	Young Adult (age 18–25) *N* = 143	Adult (age >25) *N* = 77
Endometriosis				
Age at endometriosis symptom onset, *y*, median (IQR)	14 (12–16)	13 (11–14)	14 (13–16)	15 (13–20)
Years between symptom onset and surgical diagnosis, median (IQR)	2 (1–5)	2 (1–4)	2 (1–5)	4 (1–12)
Symptoms prompting diagnosis, *n* (%)				
Pain only	339 (96.0%)	132 (97.8%)	139 (97.9%)	68 (89.5%)
Infertility or other conditions, with or without pain	14 (4.0%)	3 (2.2%)	3 (2.1%)	8 (10.5%)
Number of physicians seen before surgical diagnosis, median (IQR)	3 (2–4)	3 (2–5)	2 (2–4)	2 (1–5)
rASRM stage at surgery closest to baseline, *n* (%)				
Stage I/II	277 (94.9%)	126 (98.4%)	112 (94.9%)	39 (84.8%)
Stage III/IV	15 (5.1%)	2 (1.6%)	6 (5.1%)	7 (15.2%)
Endometriosis macrophenotype at surgery closest to baseline, *n* (%)				
Superficial only	291 (95.1%)	131 (97.0%)	118 (96.7%)	42 (85.7%)
Endometrioma	4 (1.3%)	0 (0%)	2 (1.6%)	2 (4.1%)
Deep infiltrating	10 (3.3%)	4 (3.0%)	2 (1.6%)	4 (8.2%)
Both deep infiltrating and endometrioma	1 (0.3%)	0 (0%)	0 (0%)	1 (2.0%)
Acyclic pelvic pain				
Severity of acyclic pelvic pain in the past 3 months^a^				
Median (IQR)	8 (6–9)	8 (7–9)	8 (6–9)	7 (5–9)
Mild	34 (9.8%)	11 (8.3%)	16 (11.3%)	7 (9.7%)
Moderate	72 (20.7%)	21 (15.8%)	30 (21.1%)	21 (29.2%)
Severe	241 (69.5%)	101 (75.9%)	96 (67.6%)	44 (61.1%)
Frequency of acyclic pelvic pain in the past 3 months				
Less than one day per month	36 (10.1%)	6 (4.4%)	20 (14.0%)	10 (13.0%)
Monthly but not weekly	84 (23.7%)	37 (27.4%)	30 (21.0%)	17 (22.1%)
Weekly but not daily	135 (38.0%)	50 (37.0%)	58 (40.6%)	27 (35.1%)
Daily	100 (28.2%)	42 (31.1%)	35 (24.5%)	23 (29.9%)
Acyclic pelvic pain interfered with work or school in past 3 months	236 (67.8%)	100 (75.2%)	90 (63.4%)	46 (63.0%)
Pain medications taken in the past 3 months to help with acyclic pelvic pain^b^				
None	47 (22.8%)	21 (25.3%)	19 (25.7%)	7 (14.3%)
Yes, over-the-counter medications only	95 (46.1%)	40 (48.2%)	32 (43.2%)	23 (46.9%)
Yes, prescription (with or without over-the-counter medications)	64 (31.1%)	22 (26.5%)	23 (31.1%)	19 (38.8%)
Hormonal medications used in the past 3 months to help acyclic pelvic pain^b^				
None	97 (47.1%)	39 (47.0%)	32 (43.2%)	26 (53.1%)
Yes, but pain did not get better	68 (33.0%)	29 (34.9%)	24 (32.4%)	15 (30.6%)
Yes, pain got at least somewhat better	41 (19.9%)	15 (18.1%)	18 (24.3%)	8 (16.3%)
Narcotic prescription pain medication use for 3 months or longer^b^	16 (7.5%)	4 (4.5%)	3 (3.9%)	9 (18.4%)
Traditional pain management				
Injection	4 (1.1%)	2 (1.5%)	2 (1.4%)	0 (0%)
Pain medication	220 (61.6%)	86 (62.8%)	81 (56.6%)	53 (68.8%)
TENS unit	13 (3.6%)	3 (2.2%)	8 (5.6%)	2 (2.6%)

^a^ Severity categories created using the 0 to 10 numeric rating scale (mild: 1–3, moderate: 4–6, and severe: 7–10).

^b^ Restricted to 211 participants who answered the World Endometriosis Research Foundation Endometriosis Phenome and Biobanking Harmonization.

Project compliant version of the baseline questionnaire from January 2014 onwards.

IQR, interquartile range; A2A, The Women's Health Study: From Adolescence to Adulthood.

Most participants reported using 4–8 complementary or alternative coping methods for acyclic pelvic pain (Table 3). The most common alternative method used to help with pain was the use of a heating pad (*n* = 264, 73.9%), lying down (*n* = 249, 69.7%), and sleep (*n* = 118, 46.3%), and 12.6% (*n* = 45) of all participants reported that nothing helped their pelvic/lower abdominal pain. Sleep was reported more frequently among adolescents (*n* = 59, 57.3%) compared to young adults (*n* = 40, 44.0%) and adults (*n* = 19, 31.1%; *p* = 0.004). Adolescents also reported more frequent use of music (*n* = 29, 21.2%) than young adults (*n* = 10, 7.0%) and adults (*n* = 7, 9.1%; *p* = 0.001). A greater proportion of young adults reported using laxatives/enema (*n* = 14, 9.8%) compared to adolescents (*n* = 9, 6.6%) and adults (*n* = 1, 1.3%; *p* = 0.04) for managing pain. Adults were more likely to practice yoga (*n* = 9, 14.8%) than both young adults (*n* = 10, 11.0%) and adolescents (*n* = 4, 3.9%; *p* = 0.04). These proportions were significantly different with a clear trend, however the absolute number of those practicing yoga in our study population was small.”

**Table 3:** Activities and conditions that worsen acyclic pelvic pain by age group among A2A participants who reported experiencing acyclic pelvic pain in the last 3 months at baseline (*n* = 357).

	All cases *N* = 357	Adolescent (age <18) *N* = 137	Young adult (age 18–25) *N* = 143	Adult (age >25) *N* = 77	*p*-value^a^
Total number of activities and conditions					
0	19 (5.3%)	7 (5.1%)	4 (2.8%)	8 (10.4%)	0.40
1–3	84 (23.5%)	29 (21.2%)	36 (25.2%)	19 (24.7%)	
4–8	227 (63.6%)	90 (65.7%)	92 (64.3%)	45 (58.4%)	
9 or more	27 (7.6%)	11 (8.0%)	11 (7.7%)	5 (6.5%)	
Exercise and physical activity					
Exercise	176 (49.3%)	82 (59.9%)	67 (46.9%)	27 (35.1%)	0.002
Walking	124 (34.7%)	57 (41.6%)	49 (34.3%)	18 (23.4%)	0.03
Gastrointestinal and urinary					
Bowel movement	112 (31.4%)	36 (26.3%)	44 (30.8%)	32 (41.6%)	0.07
Constipation	189 (52.9%)	75 (54.7%)	72 (50.3%)	42 (54.5%)	0.73
Full bladder	154 (43.1%)	55 (40.1%)	63 (44.1%)	36 (46.8%)	0.63
Full meal	104 (29.1%)	44 (32.1%)	42 (29.4%)	18 (23.4%)	0.40
Urination (peeing)	60 (16.8%)	23 (16.8%)	22 (15.4%)	15 (19.5%)	0.74
Sexual intercourse					
Intercourse	103 (28.9%)	10 (7.3%)	53 (37.1%)	40 (51.9%)	<0.0001
Orgasm	34 (9.5%)	6 (4.4%)	12 (8.4%)	16 (20.8%)	0.0009
Other daily activities					
Contact with clothing^b^	21 (10.0%)	12 (13.8%)	5 (6.8%)	4 (8.0%)	0.34
Coughing / sneezing	95 (26.6%)	48 (35.0%)	29 (20.3%)	18 (23.4%)	0.02
Sitting	117 (32.8%)	56 (40.9%)	41 (28.7%)	20 (26.0%)	0.04
Standing	166 (46.5%)	72 (52.6%)	65 (45.5%)	29 (37.7%)	0.11
Stress	211 (59.1%)	77 (56.2%)	99 (69.2%)	35 (45.5%)	0.002
Time of day	90 (25.2%)	40 (29.2%)	37 (25.9%)	13 (16.9%)	0.13
Weather^b^	16 (7.6%)	5 (5.7%)	5 (6.8%)	6 (12.0%)	0.43
Nothing makes my pain worse	13 (3.6%)	6 (4.4%)	3 (2.1%)	4 (5.2%)	0.36

^a^ Fisher's exact test of proportions in participants (adolescent, young adult, adult).

^b^ Restricted to 211 participants who answered the World Endometriosis Research Foundation Endometriosis Phenome and Biobanking Harmonization Project compliant version of the baseline questionnaire from January 2014 onwards.

The top activities and conditions that worsened pelvic/lower abdominal pain included stress (*n* = 211, 59.1%), constipation (*n* = 189, 52.9%), and exercise (*n* = 176, 49.3%) (Table 4). Exercise worsened pain among younger participants and was most commonly reported among adolescents (*n* = 82, 59.9%), followed by younger adults (*n* = 67, 46.9%), and adults (*n* = 27, 35.1%; *p* = 0.002). A similar trend was observed for walking specifically among adolescents (*n* = 57, 41.6%), young adults (*n* = 49, 34.3%), and adults (*n* = 18, 23.4%; *p* = 0.03). Other daily activity more commonly worsened pain among adolescents, including coughing or sneezing (*n* = 48, 35.0%) and sitting (*n* = 56, 40.9%) compared to young adults (*n* = 29, 20.3%; *n* = 41, 28.7%), and adults (*n* = 18, 23.4%, *p* = 0.02; *n* = 20, 26.0%, *p* = 0.04). Worse pain with stress was most reported by young adults (*n* = 99, 69.2%) and less common among both adolescents (*n* = 77, 56.2%) and adults (*n* = 35, 45.5%; *p* = 0.002). Lower abdominal pain was worsened by intercourse and orgasm most commonly among adults (*n* = 40, 51.9%; *n* = 16, 20.8%) compared to young adults (*n* = 53, 37.1%; *n* = 12, 8.4%), and adolescents (*n* = 10, 7.3%, *p* < 0.0001; *n* = 6, 4.4%, *p* = 0.0009). Few participants reported that nothing made their pain worse (*n* = 13, 3.6%).

**Table 4:** Complementary or alternative coping methods for managing acyclic pelvic pain by age group among A2A participants with laparoscopically confirmed endometriosis who reported experiencing acyclic pelvic pain in the last 3 months at baseline (*n* = 357).

	All cases *N* = 357	Adolescent (age <18) *N* = 137	Young Adult (age 18–25) *N* = 143	Adult (age >25) *N* = 77	*p*-value^a^
Total number of alternative methods reported					
0	29 (8.1%)	10 (7.3%)	13 (9.1%)	6 (7.8%)	0.83
1–3	110 (30.8%)	39 (28.5%)	47 (32.9%)	24 (31.2%)	
4–8	200 (56.0%)	82 (59.9%)	77 (53.8%)	41 (53.2%)	
9 or more	18 (5.0%)	6 (4.4%)	6 (4.2%)	6 (7.8%)	
Nothing helps	45 (12.6%)	17 (12.4%)	20 (14.0%)	8 (10.4%)	0.77
Gastrointestinal and urinary					
Bowel movement	94 (26.3%)	27 (19.7%)	43 (30.1%)	24 (31.2%)	0.07
Laxatives / enema	24 (6.7%)	9 (6.6%)	14 (9.8%)	1 (1.3%)	0.04
Urination (peeing)	39 (10.9%)	12 (8.8%)	18 (12.6%)	9 (11.7%)	0.58
Heat and cold					
Heating pad	264 (73.9%)	103 (75.2%)	105 (73.4%)	56 (72.7%)	0.90
Hot bath	145 (40.6%)	59 (43.1%)	52 (36.4%)	34 (44.2%)	0.41
Ice	25 (7.0%)	12 (8.8%)	8 (5.6%)	5 (6.5%)	0.57
Holistic Healing					
Massage	57 (16.0%)	17 (12.4%)	24 (16.8%)	16 (20.8%)	0.24
Acupuncture^b^	15 (5.9%)	8 (7.8%)	2 (2.2%)	5 (8.2%)	0.15
Mind-body					
Meditation	40 (11.2%)	12 (8.8%)	18 (12.6%)	10 (13.0%)	0.51
Music	46 (12.9%)	29 (21.2%)	10 (7.0%)	7 (9.1%)	0.001
Yoga^b^	23 (9.0%)	4 (3.9%)	10 (11.0%)	9 (14.8%)	0.04
Physical activity^b^	28 (11.0%)	10 (9.7%)	9 (9.9%)	9 (14.8%)	0.54
Rest and relaxation					
Lying down	249 (69.7%)	104 (75.9%)	93 (65.0%)	52 (67.5%)	0.12
Relaxation	151 (42.3%)	55 (40.1%)	63 (44.1%)	33 (42.9%)	0.80
Sleep^b^	118 (46.3%)	59 (57.3%)	40 (44.0%)	19 (31.1%)	0.004

^a^ Fisher's exact test of proportions in participants (adolescent, young adult, adult)).

^b^ These items were added to the questionnaire in May 2013 and are missing for 102 participants.

## Discussion

In our analysis of the prevalence of complementary and alternative methods used for managing acyclic pelvic pain among women living with endometriosis-associated pelvic pain, use of passive coping mechanisms, including use of heating pad and lying down, were the most commonly reported methods for pain relief. We observed significant differences in the utilization and benefit of laxatives, music, yoga, and sleep among the three age groups. Participants of all ages most reported exercise, constipation, and stress as making their acyclic pelvic pain worse.

We reported use of complementary or alternative coping methods for managing acyclic pelvic pain by age group and observed that use of a heating pad was most commonly used across all age groups. This is not surprising as heat therapy, which increases blood flow to the applied area, is a commonly suggested method for relieving tense or stiff muscles that has been used for centuries (15). However, heat therapy, including use of a heating pad, is a general recommendation and has not been well studied in the context of endometriosis-associated pain. Differences in coping methods across age groups were observed for active, mind-body practices such as yoga, meditation, and massage which were more prevalent among older women compared to adolescents. In a randomized trial of Hatha yoga, there was a reduction in the degree of daily pain among women with endometriosis, although this study excluded patients under the age of 18 (16). Holistic healing methods were more commonly reported among older women as well. A systematic review and meta-analysis of complementary treatment for women with symptomatic endometriosis found a significant benefit in reduction of pelvic pain with acupuncture compared to placebo (17, 18, 19). However, acupuncture was the least commonly used alternative method for managing pelvic/lower abdominal pain (5.9%) in our study. Women (and girls) may not be familiar with the use of acupuncture for endometriosis symptoms or may not be able to access acupuncture due to limited insurance coverage and high costs per session. It should be noted that there are also geographic, economic, and knowledge or awareness barriers to some methods, although the heterogeneity of potential barriers in our study was likely minimized since all participants were recruited from the same geographic area (Boston, MA). Lastly, we found that adolescents were almost twice as likely to have reported using sleep to manage their acyclic pelvic pain, although this may be in part due to schedule and quality of sleep differences that vary with age.

The most commonly reported activity or condition that worsened acyclic pelvic pain was stress, which was reported by all age groups. While it is well known that stress can worsen chronic pain, it remains unknown whether one may trigger or be a result of the other, which may also be unique to those who have endometriosis-associated pain (20). There were also no considerable differences in worsening of pain from any of the gastrointestinal or urinary conditions among age groups, except for a greater percentage of adults who reported that bowel movements worsened their pain (41.6%) compared to young adults (30.8%) and most dramatically, adolescents (26.3%). Development of endometriosis at an older age may be associated with a deep lesion phenotype that can involve extra pelvic locations, including the bowels (21, 22). We also observed a higher proportion of adolescents who reported exercise made their pain worse (59.9%), almost double that of adult women (35.1%). The effect of physical activity and exercise on symptoms associated with endometriosis remains somewhat unknown since previous studies vary greatly on diagnostic method for endometriosis, symptom assessment, exercise definition (23). Previous studies did not evaluate age as a potential modifier of the association between exercise and pelvic pain, although one study of physical activity during early life did not find a beneficial association between adolescent physical activity and development of endometriosis (24). However, 10%–15% of all age groups in our study reported physical activity as a coping mechanism for managing acyclic pelvic pain, further emphasizing the need for additional intervention studies of exercise with symptom management.

Regardless of age, women and girls living with endometriosis-associated pelvic pain likely use 4 or more coping methods, beyond standard hormonal or analgesic medication treatments, for pain remediation. However, utilization of these methods may also vary by age, as suggested in our analysis. While informative, the bulk of research on complementary and alternative medicine has been conducted among adult populations, and what methods are effective in this population may not apply to young adults and adolescents. For example, our study observed that 21.2% of adolescents reported music was used to manage their pelvic/lower abdominal pain, which is two-to-three times higher than among adults and young adults, respectively. Music intervention has been associated with reduced pain levels (25) and is a growing area of interest that has not been explored among endometriosis patients. Given the increased recommendation of self-management strategies for alleviating worsening endometriosis symptoms following the onset of the COVID-19 pandemic (26, 27), and that 92% of participants in our study reported using at least one alternative method for managing pelvic/lower abdominal pain, there is a clear interest and utility in integrating these methods into standard practice. In a retrospective, two-center cohort study among women with laparoscopically-confirmed endometriosis, most women reported being interested in complementary and alternative medicine (65.8%), yet few reported being well informed on these methods (10.5%) (26). Therefore, it is critical to build a more robust body of evidence that identifies complementary methods that confer sufficient benefit and those activities that may be detrimental.

Our study has several strengths, including our ability to assess complementary and alternative methods used to remediate endometriosis-associated acyclic pelvic pain reported by different age groups, including adolescents. Causes and management of pelvic pain have only been studied sparsely among adolescents, despite that this population is especially vulnerable given that adolescence is when most pelvic pain symptoms begin—and for some will have lifelong impact (1, 28). Since endometriosis can be a persistent disease, there is a high risk that symptoms will not resolve and will worsen or recur over time and it is crucial to identify methods for pain management and improvement in quality of life. We also included a comprehensive list of potential pain-managing and pain-inducing activities, allowing us to assess a variety of alternative methods in addition to exercise and physical activity. Many of the methods in this list are self-management methods, which is an important area of study since these methods are more accessible to patients and support the U.S. National Pain Strategy's recommendation to develop self-management strategies to provide consistent pain education and coping skills training (29). Furthermore, studies have shown that self-management strategies have the potential to decrease use of morphine and other opioid medications (30, 31).

However, our study is not without limitations. This was a cross-sectional assessment of multiple methods for managing acyclic pelvic pain, therefore, we cannot assume causality or conclude a specific method was effective. Although rare, our population was not inclusive of postmenopausal women who may use different coping methods to deal with ongoing or emergent pelvic pain due to endometriosis. Our assessment of yoga as a coping method was also limited since only those who were enrolled after 2013 and had the opportunity to complete the WERF EPHect compliant version of the questionnaire were asked about their use of yoga for managing acyclic pelvic pain. New complementary methods emerge over time and are subject to time trends; therefore, we cannot clarify whether lack of use for certain methods was due to ineffectiveness, lack of access, barriers to uptake, or other social factors, which may also contribute to age group variability. The questionnaires also did not assess use of herbs or supplements, including cannabis and cannabidiol, which may have benefit for relieving pelvic/lower abdominal pain (32, 33). Lastly, this study population was composed of 95% rASRM stage I/II, superficial peritoneal disease and reported pain as the only symptom prompting diagnosis, and therefore results cannot be generalized to other stages and subtypes.

In summary, our analysis of women and girls with endometriosis across the reproductive life course in the A2A cohort quantified the prevalence of complementary and alternative methods used and their reported benefit for living with non-menstrual pelvic pain. This information may be especially useful for healthcare practitioners to provide information and support for patients as they manage this highly complex and poorly understood condition (34). Future studies should aim to provide information that will further inform decisions in making care plans for managing acyclic pelvic pain, particularly endometriosis-associated pain, that is effective, accessible, and tailored to the preferences of the patient.

## Data Availability

The data analyzed in this study is subject to the following licenses/restrictions: Data are not publicly available due to information that could compromise research participants’ privacy and consent. Data requests must be reviewed and approved by the BWH Institutional Review Broad (https://www.brighamandwomens.org/research/research-administration). All inquiries should be directed to the A2A cohort leadership committee (womenshealthstudy@bwh.harvard.edu). Data sharing will require a fully executed Data Usage Agreement.
